# Recent Meeting of the American Ophthalmological Society

**Published:** 1896-10

**Authors:** John O. McReynolds

**Affiliations:** Dallas, Texas


### Recent Meeting of the American Ophthalmological Society at New London, Conn.

DALLAS, TEXAS, September 25, 1896.

*Editor Texas Medical Journal:*

A little more than two years ago I had the pleasure of attending, in London, England, a meeting of the Ophthalmological Society of the United Kingdom of Great Britain and Ireland, presided over at that time by the distinguished and courtly Argyll Robertson, of Edingburgh, and composed of such men as Jonathan Hutchinson, Nettleship, Juler, Hartridge, Frost, Tweedy, Gunn, Lang, Collins, Norton, grand old Henry Power, of London, and others. It is unnecessary to say that I was delighted at this privilege, and instructed by the papers and discussions of this meeting, and I was anxious to compare it with the body composed of the most representative oculists of our own land, the American Ophthalmological Society. But this latter privilege was reserved for me until a few weeks ago, when I was permitted to attend the annual meeting of the American Ophthalmological Society, which convened at New London, Conn. I was introduced to the society by the fluent and scholarly Henry D. Noyes, of New York, and proceeded to give my best attention the proceedings.

In conparing the British and American associations, I might refer, in passing, to a contrast in the apparel worn by the members, without meaning that in this instance the apparel proclaimed the men, for the difference was probably due to national custom rather than to elements of individual character. But at the British Association the prevailing costume was the conventional dress suit at the evening meeting, while at the American Association the main object in the mode of attire seemed to be comfort as found in the sack suit presenting the minimum degree of obstruction to the delightful breezes that floated over Long Island Sound and among the leafy trees of the Connecticut shore. The British Association met in a hall set apart permanently for such purposes, and light refreshments were served in a dining room in the same building. The American Association convened in the parlors of the Pequot House, and "table d'hote" meals were served in the main dining room of the hotel.

The British Association presented the appearance of a purely business meeting, while the American Association seemed to agreeably combine the interchange of scientific ideas with a happy relief from the busy cares of city life to some quiet retreat by the sea. The British Association devoted one entire winter evening to the hearing and discussions of a very excellent paper by Jonathan Hutchinson, Sr., on "School Ophthalmia," while the American Association presented a program of briefer papers, with more limited discussions. But both Associations seemed to be entirely free from those forms of dissipation which, I regret to say, we sometimes find associated with important and prominent conventions of medical men. A noticeable feature in the membership of the American Ophthalmological Society is that it is composed of men almost entirely from the Atlantic States, but I have never seen in any Association a finer collection of scientific men.

Among the names familiar to all we might mention Drs. Green, of St. Louis; Miller, of Providence; Prout and Allendale, of Brooklyn; Knapp, Noyes, Mittendorf, Hunter, Bull and Kaller, of New York; Harlan, Norris, Jackson, Phillips, Oliver, DeSchweinitz and Risley, of Philadelphia; Theobald, Friedenwald and Rudolph, of Baltimore; Wadsworth, Williams and Standish, of Boston.

It is not intended in this brief letter even to mention the various papers of interest presented to the society, for the proceedings will later on officially appear in due form. There was a considerable amount of original work presented, and an entire absence of tedious descriptions of well-known diseases and methods which sometimes burden the programs of medical societies, without the excuse of offering anything really new. The papers were, for the most part, clinical reports combined with observations and conclusions deduced therefrom. And the entire meeting seemed to be enjoyed by all, not only on account of its scientific features, but also on account of the social relations which the society has very properly developed.

JOHN O. McREYNOLDS, B. Sc., M. D.

